# Diagnostic performances of methylated septin9 gene, CEA, CA19-9 and platelet-to-lymphocyte ratio in colorectal cancer

**DOI:** 10.1186/s12885-024-12670-3

**Published:** 2024-07-27

**Authors:** Qian Sun, Lu Long

**Affiliations:** Department of Clinical Laboratory, Xiangya Hospital, Central South University, 87 Xiangya Road, Changsha, Hunan Province 410008 China

**Keywords:** Colorectal cancer, Methylated septin9 gene, Diagnosis, Neutrophil-to-lymphocyte ratio, Platelet-to-lymphocyte ratio

## Abstract

**Background:**

This study was designed to compare the diagnostic efficacy of mSEPT9 to four blood markers (CEA, CA19-9, platelet-lymphocyte ratio (PLR) and neutrophil-lymphocyte ratio (NLR)). In addition, we aimed to determine the combined diagnostic efficacy of mSEPT9, CEA, CA19-9, PLR and NLR in colorectal cancer.

**Methods:**

A total of 567 participants were enrolled in the study, including 308 CRC patients, 61 colorectal polyp patients and 198 healthy subjects confirmed by colonoscopy and/or tissue biopsy. Plasma samples were collected for tests.

**Results:**

The positive rate of mSEPT9 in CRC (71.8%) was markedly higher than that in either the colorectal polyps group (27.9%) or the healthy controls (6.1%) (*P* < 0.001). The levels of CEA, CA19-9, NLR and PLR in the CRC group were significantly higher than those in the non-CRC groups (*P* < 0.05). ROC curves comparison analyses showed that the diagnostic efficacy of mSEPT9 alone in CRC was significantly higher than CEA, CA19-9, NLR and PLR alone. The combination of mSEPT9 with CEA, CA19-9 and PLR showed superior diagnostic value. In addition, binary logistic regression was also used to build a better model for clinical diagnosis of CRC. On univariable analyses, age, mSEPT9, CEA, CA 19–9, PLR and NLR were independent predictors of CRC. When these covariates were fitted in multivariable models, the ones with positive detection of mSEPT9, CEA, CA 19–9 and PLR were more likely to have CRC.

**Conclusions:**

This research revealed a significant association between mSEPT9 status and the clinicopathological characteristics of CRC patients, and the combination of mSEPT9, CEA, CA19-9 and PLR could significantly improve diagnostic efficacy in CRC.

## Introduction

Colorectal cancer (CRC) ranks as the third most common malignancy and the second leading cause of cancer-related mortality worldwide, with an estimated 1.93 million new cases and 0.94 million deaths in 2020 [1]. The prevalence, incidence and death of CRC had been generally increasing from 1990 to 2019 in China. In 2019, the number of people with CRC in China was approximately 3.4 million, which was over seven times higher than that in 1990 [2]. In 2020, new CRC cases in China accounted for 28.8%, and the number of death accounted for 30.6% of all CRC globally [3]. CRC incidence has increased in recent years. It represents approximately 10% of all cancers and is the second most frequent cause of cancer deaths [4, 5]. The 5-year survival rate ranges from greater than 90% in CRC patients with stage I to slightly greater than 10% in those with stage IV [6]. Therefore, early detection of CRC is crucial to improve patient outcomes. Despite recent achievements in the diagnosis of CRC, colonoscopy plus pathological examination is still considered the gold standard. However, its compliance rate remains very low due to its invasiveness, expense, dietary restriction requirement, and extensive bowel preparation [7]. Thus, a noninvasive, highly accurate screening method to detect CRC at an early stage is urgently needed, especially for those who are reluctant to undergo colonoscopy examinations. In China, although various noninvasive methods have been applied, there is no ideal diagnostic assay for the detection of CRC thus far.

DNA methylation is an important epigenetic modification, and CpG islands are the main site of DNA methylation and are closely related to the occurrence and development of tumours [8]. CpG islands can be successfully detected in several types of biological samples (blood, tissue, stool) [9, 10]. Due to their biological rationality and user-friendly nature, DNA methylation-based biomarkers are valuable tools in the early detection of CRC [11]. Among various methylated genes, the methylated septin9 gene (mSEPT9) has been found to have extremely high methylation levels in colorectal tumour tissues [12] and has been studied for serological diagnosis of CRC with high sensitivity [13, 14]. The septin9 gene is a member of a highly conserved cytoskeletal protein family with guanosine triphosphatase activity, which plays an important role in a variety of biological functions, including division, polarization, apoptosis, and so on [15]. mSEPT9 testing is the first blood test approved by the FDA for colorectal cancer screening.

Inflammation plays an important role in the occurrence and development of many kinds of malignant tumours. Related indicators of the inflammatory response, such as the neutrophil-lymphocyte ratio (NLR) and the platelet-lymphocyte ratio (PLR), have been widely used in the diagnosis and prognosis of cancer patients [16]. Serum tumour markers, such as carcinoembryonic antigen (CEA) and carbohydrate antigen 19 − 9 (CA19-9), are widely used in the early diagnosis and prognosis of colorectal cancer, but the sensitivity and specificity for early CRC are unsatisfactory [17]. To date, no previous studies have compared the performance of mSEPT9 testing with that of each of these serum markers.

In this study, mSEPT9 and tumour markers CEA, CA125, and CA19-9 as well as common blood examination indices such as NLR and PLR were analysed to explore the value of single and combined detection of these indices in the early diagnosis of colorectal cancer to provide a reliable clinical basis for the early clinical diagnosis of colorectal cancer. This study may provide some valuable information for the screening and diagnosis monitoring of CRC, especially in those patients for whom it is difficult to obtain biopsy specimens or who are not willing to undergo intestinal preparation.

## Materials and methods

### Study subjects

**Fig. 1:**
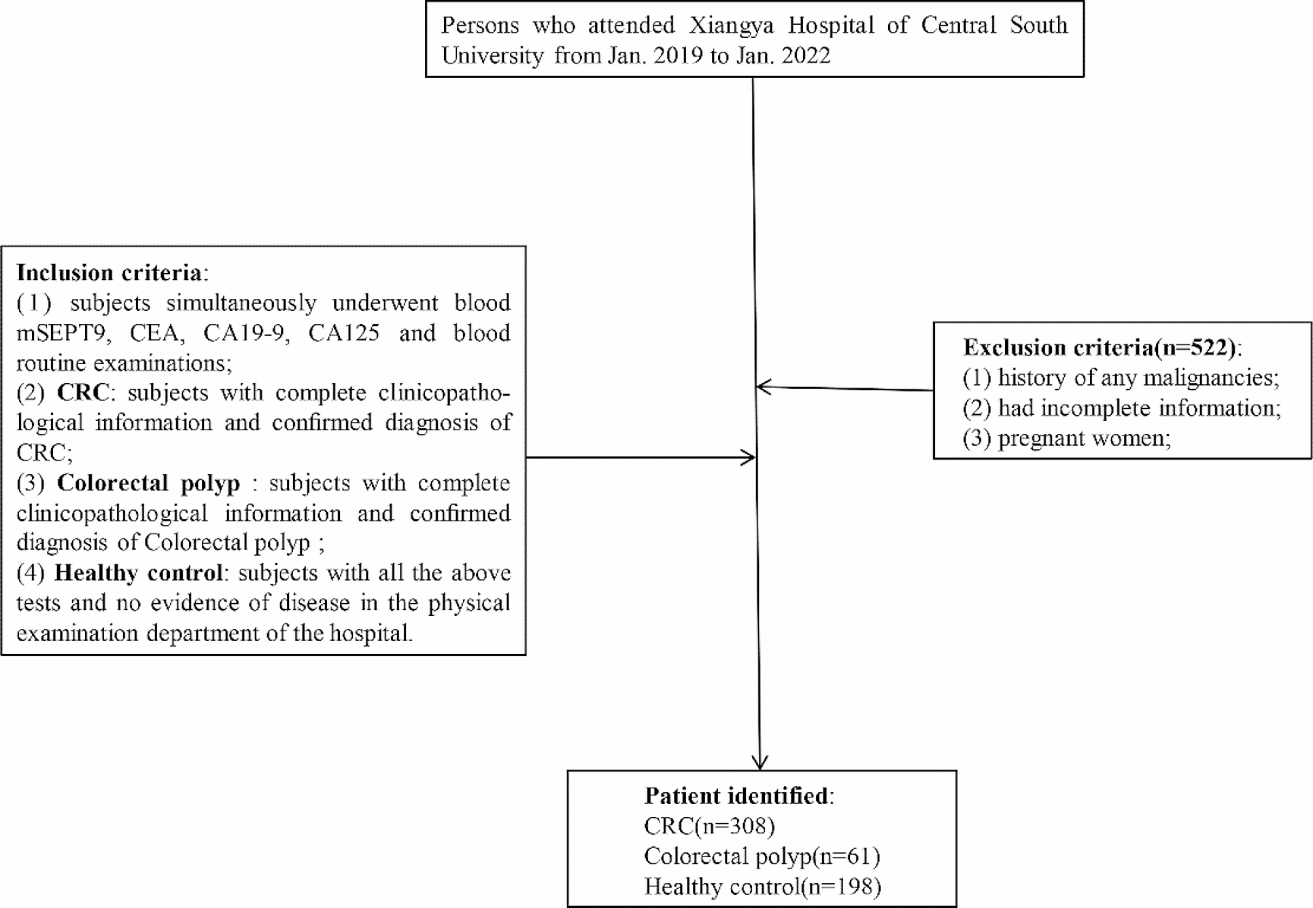
Flow chart depicting patient selection

We retrospectively analysed data from 308 primary CRC patients, 61 colorectal polyp patients and 198 healthy subjects at Xiangya Hospital of Central South University from Jan. 2019 to Jan. 2022. All participants signed written informed consent, and their final diagnosis was determined based on the results of colonoscopy and/or histological testing. The procedure of patient selection was depicted in a flowchart in Fig. 1. All enrolled subjects simultaneously underwent blood mSEPT9, CEA, CA19-9, CA125 and blood routine examinations. The main exclusion criteria were a history of any malignancies, pregnancy, and incomplete information. The demographic and clinicopathological information of the subjects, including sex, age, cancer stage, pathological type, tumour differentiation, primary tumour (T) categories, regional node (N) categories, distant metastasis categories (M), vascular invasion (V), and tumour location, was collected. Tumour stages were defined according to the tumour-node-metastasis staging system of the 8th edition of the Cancer Staging Manual of the American Joint Committee on Cancer [18]. This study was approved by the ethics committee of Xiangya Hospital of Central South University. Due to the retrospective character of this study and concealment of patient information, the Ethics Committee of Xiangya Hospital of Central South University waived off the requirement for informed consent from the study subjects for the use of their data.

### Methylated septin9 gene detection

A 10 mL peripheral blood sample was collected with a K2EDTA anticoagulant tube for the methylated septin9 gene (mSEPT9) assay. The plasma was separated from the blood sample at 1350 g for 12 min within 2 h. The plasma sample was immediately tested or stored at -20 ± 5℃ for no more than 1 week. The mSEPT9 assay was performed with the septin9 Gene Methylation Detection Kit (Beijing BioChain Co., Ltd., China). First, DNA extraction and bisulfite conversion of plasma samples were performed following the manufacturer’s instructions. Then, polymerase chain reaction was used to detect mSEPT9 fragments and the internal control ACTB, which was used to assess whether the amount of DNA in the test was sufficient. Real-time PCR amplification for methylated detection of the septin9 gene was run on an ABI 7500 real-time PCR system (Thermo Fisher Scientific, MA, USA), and the PCR procedure was initiated at 94℃ for 20 min, followed by 45 cycles at 62℃ for 5 s, 55.5℃ for 35 s, 93℃ for 30 s, and finally 40℃ for 5 s. Both positive and negative controls were tested in each of the reactions. The methylation results were strictly judged in accordance with the manufacturer’s instructions. The results were considered valid when the cycle threshold (Ct) value of ACTB was less than or equal to 32, and the negative and positive controls met the validity criteria specified by the manufacturer. Patients with a Ct value of mSEPT9 less than or equal to 41 were considered to be in the mSEPT9-positive group, whereas those with a Ct value over 41 or not detected were considered to be in the mSEPT9-negative group.

### CEA, CA19-9 and CA125 levels

A total of 3–5 ml of venous blood was collected. Serum was isolated via centrifugation at 3,000 rpm for 5 min. The serum levels of CEA, CA19-9 and CA125 were detected using a SLXO-001 automatic biochip reader and the original matching reagents (Sanlian Biological Co., Ltd., Jiangsu, China). The principle is a microarray chemiluminescence immunoassay. All negative controls, low levels of positive controls and high levels of positive controls were tested in each detection. The cutoff value for normal CEA was < 5 ng/mL and that for normal CA19-9 and CA125 were both < 35 U/mL according to the manufacturer’s instructions.

### NLR and PLR levels

Venous blood **(**3–5 ml) was collected in the morning, and routine blood tests were carried out by using a Beckman Coulter DxH 800 blood cell analyser and the corresponding reagents from the original factory. The neutrophil, lymphocyte, and platelet counts were obtained, and the NLR and PLR were calculated.

### Statistical analysis

Statistical analysis was performed using SPSS 27.0 software (SPSS Inc., IL, USA) or GraphPad Prism version 6.0 (GraphPad Software, San Diego, CA, USA). Data were tested for normality using the Kolmogorov–Smirnov test. Descriptive statistics are presented for each group as medians (interquartile ranges; IQRs) for continuous variables and as numbers (percentages) for categorical data. Comparisons of different groups were performed using the Mann–Whitney U test or the Kruskal‒Wallis H test for continuous variables and χ^2^ or Fisher’s exact test for categorical variables. To evaluate the value of the blood mSEPT9 assay in the diagnosis of CRC, we plotted the receiver operating characteristic (ROC) curves for CRC compared with CEA, CA19-9, NLR and PLR. Binary univariable logistic regression analysis was used to assess the associations between subjects’ characteristics and the risk of CRC. Then all variables tested in univariate logistic regression with a *P* value < 0.05 were tested in multivariate logistic regression. All *P* values were 2-sided, and *P* < 0.05 was considered statistically significant.

## Results

### Clinical characteristics of participants

In total, 567 participants were enrolled in the study, including 335 males (59.1%) and 232 females (40.9%). There were 308 cases of CRC, 61 of colorectal polyps, and 198 healthy persons confirmed by colonoscopy and/or tissue biopsy. The numbers of subjects in the CRC, colorectal polyp and healthy control groups were 308 (176 male, 132 female, median age 57 years), 61 (39 male, 22 female, median age 56 years) and 198 (120 male, 78 female, median age 55 years), respectively. There was no significant difference in sex composition among the groups. The clinical characteristics of the participants are shown in Table 1. In the CRC group, 137 (44.5%), 166 (53.9%) and 5 (1.6%) patients were diagnosed with colon cancer, rectum and rectosigmoid transition cancer, respectively. Within the colon cancer case group, tumour locations in the sigmoid colon constituted the largest proportion at 41.6% (57/137), compared with tumour locations in the ascending colon at 18.2% (25/137), transverse colon at 16.1% (22/137), descending colon at 6.6% (9/137), and unspecified colon at 17.5% (24/137). The numbers of CRC patients in stage I, stage II, stage III, and stage IV were 43 (14.0%), 81 (26.3%), 166 (53.9%), and 18 (5.8%), respectively. The levels of CEA, CA19-9, lymphocytes, PLT, NLR and PLR in the CRC group were significantly higher than those in the colorectal polyps group and healthy controls (*P* < 0.05), and no significant association was found between the colorectal polyps group and healthy controls.

**Table 1:** Demographic and clinical statistics of the study subjects in different groups (*N*=567)

Parameters	Overall	CRC	Colorectal polyps	Healthy control	*P*
**Number**	567	308	61	198	
**Sex**, **n (%)**					0.532
Female	232(40.9)	132 (42.9)	22 (36.1)	78(39.4)	
Male	335 (59.1)	176(57.1)	39(63.9)	120(60.6)	
**Age**, **median**, **(years)**	56(50-64)	57(51-65)	56(49-68)	55(48-62)	<0.001^**^
<60, n (%)	343(60.5)	177(57.5)	37(60.7)	129(65.2)	
≥60, n (%)	224(39.5)	131(42.5)	24(39.3)	69(34.8)	
**Cancer stage**					
I		43(14.0)			
II		81(26.3)			
III		166(53.9)			
IV		18(5.8)			
**Tumour location**					
Rectum		166(53.9)			
colon		137(44.5)			
Rectosigmoid transition		5(1.6)			
**Cancer differentiation**					
High		16(5.2)			
Moderate		273(88.6)			
Low		19(6.2)			
**Vascular infiltration**					
Absent		213(69.2)			
Present		66(21.4)			
Unknown		29(9.4)			
**Neural infiltration**					
Absent		228(74.0)			
Present		51(16.6)			
Unknown		29(9.4)			
**Tumour size(cm)**					
<5		186(60.4)			
≥5		90(29.2)			
Unknown		32(10.4)			
**Cancer type**					
Protrude		70(22.7)			
Ulcerative		181(58.8)			
Unknown		57(18.5)			
**Tumour markers**					
CEA, ng/ml		2.86(1.34-7.66)	1.39(0.94-2.17)	1.02(0.63-1.58)	<0.001^**^
CA19-9, U/ml		11.69(6.15-25.34)	8.22(3.58-11.17)	6.23(3.33-11.13)	<0.001^**^
CA125, U/ml		7.93(5.56-13.44)	7.51(6.12-9.83)	7.23(5.80-9.67)	0.035^*^
**Inflammation marker**					
Neutrophil, ×10^9^ /L		3.40(2.60-4.10)	3.10(2.70-3.90)	3.20(2.60-4.00)	0.432
Lymphocyte, ×10^9^/L		1.40(1.10-1.70)	1.70(1.30-1.90)	1.70(1.40-2.10)	<0.001^**^
PLT, ×10^9^/L		223.50(187.00-273.75)	206.00(183.00-238.00)	206.50(175.00-237.00)	<0.001^**^
**NLR**		2.45(1.92-3.00)	1.93(1.60-2.43)	1.95(1.51-2.33)	<0.001^**^
**PLR**		161.46(128.22-212.56)	127.22(108.50-144.29)	119.46(101.51-145.70)	<0.001^**^

Note: Data are presented as the number of patients or median (Q25–Q75). Differences between groups were assessed with the Kruskal‒Wallis H test. A p value < 0.05 was used to indicate a statistically significant result (**p< 0.001, *p< 0.05)

Abbreviations: CEA, carcinoembryonic antigen; CA19-9, carbohydrate antigen 19-9; CA125, carbohydrate antigen 125; PLT, platelet; NLR, neutrophil-lymphocyte ratio; PLR, platelet-lymphocyte ratio

### Positive detection rates of mSEPT9

**Fig. 2:**
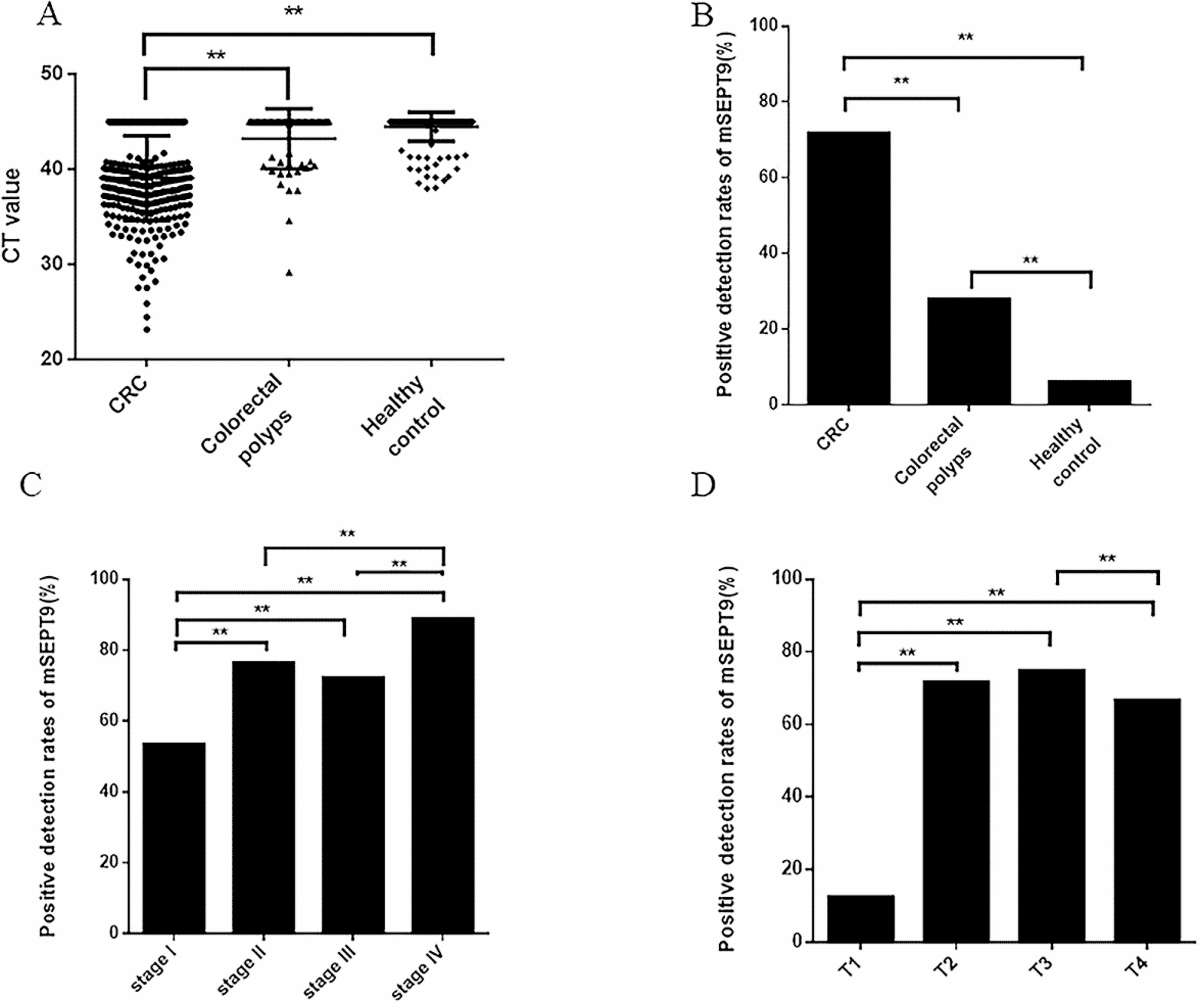
Positive detection rates of mSEPT9. (**A**) Levels of methylated septin9 in the plasma of patients with CRC, colorectal polyps, and healthy controls; (**B**) The positive detection rates of mSEPT9 in CRC, colorectal polyps, and the healthy control group; (**C**) The positive detection rates of mSEPT9 in CRC patients at different cancer stages; (**D**) The positive detection rates of mSEPT9 in CRC patients at different primary tumour (T) stages; **P< 0.001

Quantitative RT‒PCR analysis was performed to detect mSEPT9 in participants’ plasma samples. As shown in Fig. 2A and B, the septin9 methylation levels in the CRC group were significantly higher than those in the colorectal polyps group and healthy controls (*P* < 0.001). The positive rate of mSEPT9 in CRC (71.8%) was markedly higher than that in either the colorectal polyps group (27.9%) or the healthy controls (6.1%) (*P* < 0.001). After determining the performance of the plasma mSEPT9 assay for evaluating CRC, we further explored the correlation between mSEPT9 status and clinicopathological characteristics. As shown in Table 2, the positive detection rate of mSEPT9 among CRC patients older than 60 years (74.8%) was similar to that among those younger than 60 years (69.5%, *P* = 0.305). The positivity rate of mSEPT9 was significantly higher in male patients (76.7%) than in female patients (65.2%, *P* = 0.026). Moreover, the positivity rate of mSEPT9 was significantly higher in patients with more advanced TNM stages (stage I: 53.5%, stage II: 76.5%, stage III: 72.3%, stage IV: 88.9%, *P* = 0.014) than in patients with less advanced stages (Fig. 2C). Further analysis showed that mSEPT9 positivity was also significantly greater in patients with a more advanced T stage (stage T1: 12.5%, stage T2: 71.7%, stage T3: 74.8%, stage T4: 66.7%, *P* = 0.002) than in patients with less advanced stages (Fig. 2D), although there was no significant relationship between mSEPT9 status and N stage (*P* = 0.075) or M stage (*P* = 0.096). However, no significant association was found between the mSEPT9 positivity rate and age, cancer differentiation, lymph node metastasis, vascular invasion, nerve invasion, tumour size, or cancer type.

**Table 2:** Association between the positive detection rate of *mSEPT9* and demographic and clinical statistics of patients with CRC [n (%)]

Parameters	Overall	mSEPT9-positive cases	mSEPT9-negative cases	*P*
**Number**	308	221(71.8)	87(28.2)	
**Sex**				0.026^*^
Female	132	86 (65.2)	46(34.8)	
Male	176	135 (76.7)	39(23.3)	
**Age**				0.305
<60	177	123(69.5)	54(30.5)	
≥60	131	98(74.8)	23(25.2)	
**Cancer stage**				0.014^*^
I	43	23(53.5)	20(46.5)	
II	81	62(76.5)	19(23.5)	
III	166	120(72.3)	46(27.7)	
IV	18	16(88.9)	2(11.1)	
**Tumour location**				0.019^*^
Rectum	166	108(65.1)	58(34.9)	
colon	137	109(79.6)	28(20.4)	
**Rectosigmoid transition**	5	4(80.0)	1(20.0)	
**Primary tumour(T) stage**				0.002^*^
T1	8	1(12.5)	7(87.5)	
T2	60	43(71.7)	17(28.3)	
T3	210	157(74.8)	53(25.2)	
T4	30	20(66.7)	10(33.3)	
**lymph node(N) metastasis**				0.075
N0	124	84(67.7)	40(32.3)	
N1	112	89(79.5)	23(20.5)	
N2	72	48(66.7)	24(33.3)	
**Distant metastasis(M)**				0.096
M0 or MX	290	205(70.7)	85(29.3)	
M1	18	16(88.9)	2(11.1)	
**Cancer differentiation**				0.656
High	16	11(68.8)	5(31.2)	
Moderate	273	198(72.5)	75(27.5)	
Low	19	12(63.2)	7(36.8)	
**Vascular infiltration**				0.931
Absent	213	153(71.8)	60(28.2)	
Present	66	48(72.7)	18(27.3)	
Unknown	29	20(69.0)	9(31.0)	
**Neural infiltration**				0.322
Absent	228	160(70.2)	68(29.8)	
Present	51	41(80.4)	10(19.6)	
Unknown	29	20(69.0)	9(31.0)	
**Tumour size(cm)**				0.079
<5	186	125(67.2)	61(32.8)	
≥5	90	72(80.0)	18(20.0)	
Unknown	32	24(75.0)	8(25.0)	
**Cancer type**				0.112
Protrude	70	46(65.7)	24(34.3)	
Ulcerative	181	138(76.2)	43(23.8)	
Unknown	57	37(64.9)	20(35.1)	

Note: A *p* value < 0.05 was used to indicate a statistically significant result (^*^p< 0.05)

### Associations between other blood markers and clinicopathological parameters in the CRC group

The associations between the biomarkers and clinical parameters were analysed in the CRC groups (Table 3). There was no significant difference found in the levels of CEA, CA19-9, NLR and PLR among different cancer differentiations and locations. The level of CEA was significantly higher in patients with more advanced TNM stages than in patients with less advanced stages (*P* < 0.001), which was also significantly greater in patients with a more advanced T stage (*P* < 0.001), N stage (*P* = 0.003) and M stage (*P* = 0.001) than in patients with less advanced stages. The PLR was significantly higher in female patients and patients younger than 60 years than in male patients and patients older than 60 years (*P* = 0.017).

**Table 3:** Association between other blood markers and demographic and clinical statistics of patients with CRC

Parameters	CEA	*P*	CA19-9	*P*	NLR	*P*	PLR	*P*
**Sex**								
Female	2.20(1.01-5.46)	0.04^*^	11.17(6.24-21.74)	0.308	2.31(1.76-3.00)	0.146	174.73(137.80-230.76)	0.007^*^
Male	3.600(1.625-9.51)		12.00(6.15-31.83)		2.53(2.00-3.00)		155.00(121.55-202.39)	
**Age**								
<60	2.69(1.22-8.29)	0.983	12.06(6.07-29.09)	0.406	2.43(1.84-3.00)	0.411	169.09(133.57-222.14)	0.017^*^
≥60	3.26(1.44-6.55)		10.61(6.215-22.37)		2.50(2.00-3.04)		155.00(124.69-190.00)	
**Cancer stage**								
I	1.87(0.71-3.02)	<0.001^**^	9.97(6.38-19.89)	0.068	2.29(1.92-2.72)	0.680	160.59(137.43-187.31)	0.067
II	3.06(1.22-7.78)		9.47(4.89-18.75)		2.60(1.93-3.23)		182.22(135.38-260.00)	
III	3.090(1.605-8.205)		12.93(6.73-29.75)		2.41(1.87-3.00)		157.21(121.99-205.42)	
IV	42.18(3.65-147.18)		15.43(6.39-122.03)		2.59(2.16-2.91)		159.34(132.60-185.59)	
**Tumour location**								
Rectum	2.58(1.16-7.55)	0.090	10.53(6.33-24.75)	0.663	2.45(1.93-3.00)	0.202	157.88(125.83-205.29)	0.104
colon	2.94(1.43-7.56)		12.79(6.23-24.90)		2.43(1.92-2.92)		170.00(133.85-215.83)	
**Rectosigmoid transition**	195.54(4.44-433.11)		5.02(1.60-35.59)		2.64(2.58-4.64)		187.27(160.71-236.36)	
**T stage**								
T1	1.74(0.71-3.13)	<0.001^**^	7.99(7.20-12.71)	0.795	2.82(2.02-3.20)	0.848	173.22(150.78-183.57)	0.814
T2	1.98(0.85-4.42)		12.09(6.36-23.98)		2.34(1.89-2.90)		155.84(126.32-190.40)	
T3	3.42(1.57-9.20)		11.53(6.01-24.86)		2.50(1.92-3.06)		162.82(127.77-216.28)	
T4	3.85(1.77-10.12)		13.73(6.32-65.64)		2.48(2.14-2.85)		165.85(134.39-214.00)	
**N stage**								
N0	2.27(1.00-4.74)	0.003^*^	9.63(5.44-18.41)	0.007^*^	2.45(1.92-3.02)	0.176	174.65(135.93-225.83)	0.187
N1	2.70(1.41-7.91)		12.40(6.31-33.72)		2.33(1.84-2.94)		154.43(120.40-209.86)	
N2	4.89(1.89-11.46)		14.51(8.70-36.34)		2.55(2.10-3.06)		161.46(126.63-197.72)	
**M stage**								
M0 or MX	2.685(1.312-6.960)	0.001^*^	11.645(6.138-24.365)	0.323	2.441(1.905-3.000)	0.676	162.250(127.670-214.222)	0.722
M1	42.175(3.650-147.183)		15.425(6.388-122.028)		2.590(2.158-2.910)		159.335(132.595-185.585)	
**Cancer differentiation**								
High	4.85(1.60-8.53)	0.821	15.53(6.97-37.65)	0.517	2.11(1.70-2.64)	0.068	155.39(126.06-191.23)	0.105
Moderate	2.81(1.32-7.78)		11.61(6.13-26.64)		2.45(1.92-3.00)		160.71(126.67-211.76)	
Low	2.84(1.62-6.06)		9.92(6.12-15.58)		2.71(2.14-3.69)		195.63(160.16-243.57)	

Note: Data are presented as positive detection rate % or median (Q25–Q75). A *p* value < 0.05 was used to indicate a statistically significant result (^**^p< 0.001, ^*^p< 0.05)

### Diagnostic value of mSEPT9 and other blood markers for CRC

**Fig. 3:**
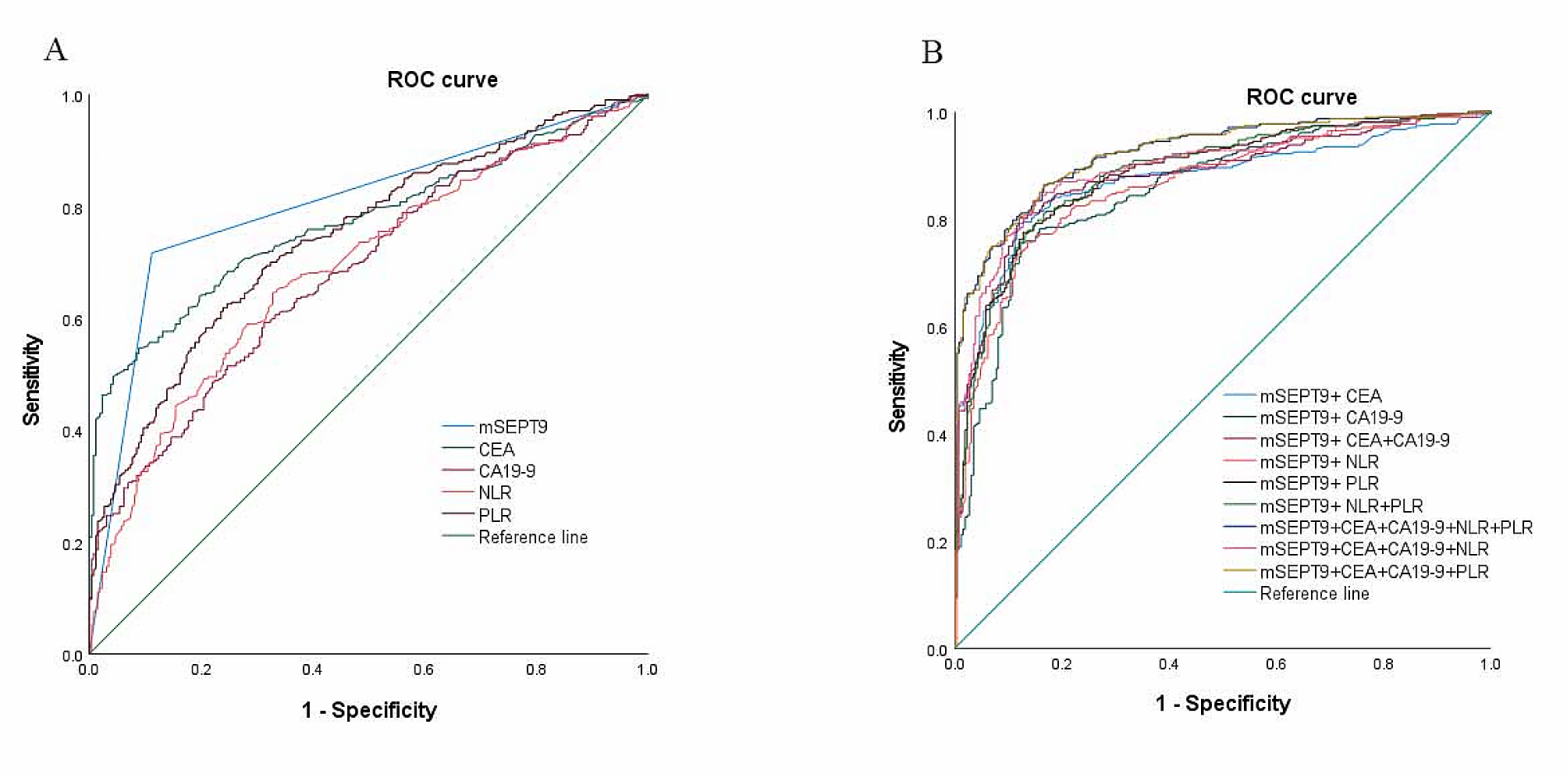
Receiver operating characteristic curves for mSEPT9, CEA, CA19-9, NLR, PLR and the combination. (**A**) The diagnostic values of mSEPT9, CEA, CA19-9, NLR, and PLR for detecting colorectal cancer; (**B**) The diagnostic values of the combination of mSEPT9 with CEA, CA19-9, NLR and/or PLR for detecting colorectal cancer

ROC curve was used to evaluate the efficacy of single detection and combined detection in CRC diagnosis. As shown in Table 4; Fig. 3, the areas under the ROC curve (AUCs) for mSEPT9, CEA, CA19-9, NLR and PLR as parameters in the diagnosis of CRC were 0.802, 0.771, 0.685, 0.690 and 0.744, respectively. At the cutoff value of 41.0 for mSEPT9, we distinguished patients with CRC from non-CRC patients with a sensitivity of 71.7% and a specificity of 88.8%. Notably, the diagnostic performance of CEA and CA19-9 could be improved when mSEPT9, CEA and CA19-9 were combined for detection. The AUCs for mSEPT9 + CEA and mSEPT9 + CA19 − 9 were 0.804 and 0.857, respectively (Fig. 3B; Table 4), which were significantly higher than those for CEA (0.771) or CA19 − 9. Meanwhile, the diagnostic performance of NLR and PLR could also be improved when mSEPT9, NLR and PLR were combined for detection. The AUCs for mSEPT9 + NLR and mSEPT9 + PLR were 0.862 and 0.885, respectively, which were significantly higher than those for NLR (0.690) or PLR (0.744). Moreover, the sensitivity of mSEPT9 + NLR + PLR (81.8%) was significantly higher than that of NLR (64.5%) + PLR (61.9%). When the five markers were combined, the AUC, sensitivity, and specificity of mSEPT9 + CEA + CA19 − 9 + NLR + PLR were 0.923 (95% CI: 0.901–0.944), 86.3%, and 83.3%, respectively. Moreover, we were surprised to find that the AUC, sensitivity, and specificity of mSEPT9 + CEA + CA19 − 9 + PLR was almost the same as the five markers (mSEPT9 + CEA + CA19 − 9 + NLR + PLR) combined. Therefore, these results suggested that circulating mSEPT9 may represent a promising biomarker for CRC. The detection of mSEPT9, CEA, CA19-9 and PLR may provide better diagnostic performance in discriminating patients with CRC from non-CRC individuals, with higher sensitivity and specificity. In addition, binary logistic regression was also used to build a better model for clinical diagnosis of CRC. On univariable analyses, age, mSEPT9, CEA, CA 19–9, PLR and NLR were independent predictors of CRC (all *P* < 0.01; Table 5). When these covariates were fitted in multivariable models, the ones with positive detection of mSEPT9, CEA, CA 19–9 and PLR were more likely to have CRC (all *P* < 0.05) (Table V).

**Table 4:** Diagnostic value of *mSEPT9* and other blood markers for detecting colorectal cancer

	AUC (95% CI)	Sensitivity	Specificity	*P*
mSEPT9	0.802(0.765-0.840)	0.717	0.888	<0.001^**^
CEA	0.771(0.733-0.810)	0.547	0.911	<0.001^**^
CA19-9	0.685(0.641-0.728)	0.590	0.687	<0.001^**^
NLR	0.690(0.647-0.733)	0.645	0.672	<0.001^**^
PLR	0.744(0.704-0.784)	0.619	0.760	<0.001^**^
mSEPT9+ CEA	0.804(0.842-0.902)	0.792	0.880	<0.001^**^
mSEPT9+ CA19-9	0.857(0.826-0.888)	0.756	0.880	<0.001^**^
mSEPT9+ CEA+CA19-9	0.881(0.852-0.909)	0.801	0.880	<0.001^**^
mSEPT9+NLR	0.862(0.831-0.892)	0.756	0.865	<0.001^**^
mSEPT9+PLR	0.885(0.857-0.912)	0.775	0.872	<0.001^**^
mSEPT9+NLR+ PLR	0.886(0.859-0.913)	0.811	0.833	<0.001^**^
mSEPT9+CEA+CA19-9+NLR	0.896(0.869-0.922)	0.844	0.842	<0.001^**^
mSEPT9+CEA+CA19-9+ PLR	0.923(0.901-0.944)	0.863	0.834	<0.001^**^
mSEPT9+CEA+CA19-9+NLR+ PLR	0.923(0.901-0.944)	0.863	0.833	<0.001^**^

Note: A *p* value < 0.05 was used to indicate a statistically significant result (**p< 0.001)

**Table 5:** Binary logistic regression on risk factors for CRC

	Variable	*R* ^2^	SE	Waldχ^2^	*P*	OR	95%CI
Univariate binary logistic regression	Age	0.021	0.008	7.256	0.007	1.002	1.006~1.038
	mSEPT9	3.003	0.234	164.417	<0.001	20.147	12.731~31.883
	CEA	0.611	0.079	59.383	<0.001	1.842	1.577~2.151
	CA 19–9	0.054	0.009	35.115	<0.001	1.055	1.037~1.074
	PLR	0.019	0.002	71.755	<0.001	1.019	1.015~1.024
	NLR	0.682	0.117	33.993	<0.001	1.978	1.573~2.489
Multivariate binary logistic regression	mSEPT9	2.472	0.274	81.670	<0.001	11.851	66.932~20.259
	CEA	0.411	0.087	22.507	<0.001	1.509	1.273~1.788
	CA 19–9	0.025	0.012	4.373	0.037	1.026	1.002~1.050
	PLR	0.019	0.495	46.747	<0.001	1.019	1.014~1.025

## Discussion

CRC is a common gastrointestinal malignant tumour of the digestive tract with a high mortality rate and poor prognosis, for which early detection and early diagnosis are very efficient strategies to reduce the morbidity and mortality of CRC, especially in high-risk populations. Blood-based tests have the advantage of minimal invasiveness compared to invasive colonoscopy, and they are anticipated to have higher compliance rates than stool-based tests [19]. However, the specificity and sensitivity of two traditional blood-based tumour biomarkers (CEA and CA19-9) have been evaluated to be low, especially for stratifying early stages of CRC [20, 21], which was further confirmed in our study. Growing evidence has suggested that DNA methylation may play a role in driving the occurrence of CRC [22, 23]. An increasing number of genes with methylation have been proven to be involved in the tumorigenesis of CRC [24]. Investigations on blood-based biomarkers for early detection of CRC are highly warranted because stool-based tests are not convenient. mSEPT9, as a specific molecular-level tumour marker of CRC, is organ-specific and is associated with better compliance [25, 26], providing a promising supplement to traditional laboratory-assisted diagnostic methods. In this study, we evaluated the diagnostic value of mSEPT9 for blood-based CRC detection in Central South Chinese patients compared with CEA and CA19-9 and two common blood examination indices (NLR and PLR). Our findings showed that mSEPT9 performed better than other blood markers (CEA, CA19-9, NLR and PLR) for CRC diagnosis, in which patients with CRC in China were distinguished from healthy individuals with a sensitivity of 71.7%, specificity of 88.8%, and AUC of 0.802. The positive rate of mSEPT9 in CRC (71.8%) was markedly higher than that in either the colorectal polyps group (27.9%) or the healthy controls (6.1%), and these findings were consistent with those of previous studies [27, 28].

In our studies, we investigated the association between mSEPT9 status and the clinicopathological characteristics of patients with CRC. We found that mSEPT9 can be detected at all stages and that the positive rates of mSEPT9 were significantly associated with TNM stage. The positivity rate of mSEPT9 was significantly higher in patients with more advanced TNM stages (stage I: 53.5%, stage II: 76.5%, stage III: 72.3%, stage IV: 88.9%) than in patients with less advanced stages. The sensitivity was highest (88.9%) for stage IV disease, consistent with a previous study that showed a higher sensitivity for patients with stage II–IV disease [29]. It means that mSEPT9 appeared to have higher detection efficiency for late-stage CRC compared with early-stage ones. Furthermore, mSEPT9 performed outstandingly as an auxiliary molecular staging parameter [30] and the degree of DNA methylation has been proved increasing with advanced CRC-stages [31]. Further analysis showed that mSEPT9 positivity was also significantly greater in patients with a more advanced T stage (stage T1: 12.5%, stage T2: 71.7%, stage T3: 74.8%, stage T4: 66.7%) than in patients with less advanced stages, although there was no significant relationship between mSEPT9 status and N stage or M stage. These findings were consistent with those of Sun et al [27]. However, a previous study by Fu et al [28]. on 98 CRC cases showed no significant association between mSEPT9 and TNM stage, which may be caused by a relatively smaller sample size. Our findings demonstrate that CRC patients with advanced-stage disease are more easily detected by mSEPT9 than those with early-stage disease. The level of CEA was significantly higher in patients with more advanced TNM stages than in patients with less advanced stages and was also significantly greater in patients with a more advanced T stage, N stage and M stage than in patients with less advanced stages. However, there was no significant difference found in the levels of CA19-9, NLR or PLR among different cancer stages.

The systemic inflammatory response accompanies the development of cancer, whether early or advanced cancer, which provides us with new methods for the early identification of CRC [32, 33]. The NLR and PLR, which are markers of systemic inflammation, are expected to aid the early diagnosis of CRC. In our study, we showed that NLR and PLR could act as early diagnostic markers for CRC which was in line with Jia’s study [34] and were also proved to be associated with the progression of CRC, which still need to be confirmed in further studies. In the present study, it was found that the systemic inflammatory markers NLR and PLR were similarly valuable for the diagnosis of GC as the traditional tumour markers CEA and CA19-9. The detection sensitivity of mSEPT9 for CRC reached 71.7% among the included individuals, which was consistent with a previous study showing 76.4% [35]. When compared with other blood markers, such as CEA (AUC: 0.771,95%CI:0.733–0.810), CA19–9 (AUC: 0.685,95%CI:0.641–0.728), NLR (AUC: 0.690,95%CI:0.647–0.733), and PLR (AUC: 0.744,95%CI: 0.704–0.784), mSEPT9 with an AUC of 0.802(95%CI:0.765–0.840) showed a better diagnostic efficiency, consistent with a previous study [36, 37], indicating that mSEPT9 is specifically elevated in CRC patients and can be used as a tumour marker for CRC diagnosis. To the best of our knowledge, no previous study has reported the combined diagnostic efficacy of mSEPT9, CEA, CA19-9, NLR and PLR for CRC. In this study, we found that the diagnostic performance of NLR and PLR could be improved upon when mSEPT9, NLR and PLR were combined for detection. The AUCs for mSEPT9 + NLR and mSEPT9 + PLR were 0.862(95%CI:0.831–0.892) and 0.885(95%CI:0.857–0.912), respectively. Meanwhile, the sensitivity of mSEPT9 + NLR + PLR (81.8%) was significantly higher than that of NLR (64.5%) + PLR (61.9%). We found that the combination of mSEPT9 with CEA, CA19-9 and PLR showed superior diagnostic value. The AUC further improved to 0.923(95%CI: 0.901–0.944), and the sensitivity improved to 86.3%. In addition, to confirm the optimization of this combination, we also used binary univariable and multivariable logistic regressions for the selection of predictors. The same biomarkers, mSEPT9, CEA, CA19-9 and PLR were finally selected to construct the CRC diagnostic model for predicting the risk of CRC.

Taken together, these findings suggested that mSEPT9 could be a novel promising biomarker for CRC detection. The detection of mSEPT9, CEA, CA19-9 and PLR may provide better diagnostic performance in discriminating patients with CRC from non-CRC individuals. Despite the novel and clinically relevant findings in this study, there are some limitations. First, the short follow-up duration in this study with a single-centre retrospective design may provide bias towards sample selection and analysis. At the same time, all patients in our study were Chinese subjects; thus, racial differences should be noted when applying the conclusions to other populations. Second, our sample size for the study may not be large enough for the stratified analysis of cancer patients with different stages and different locations, and a larger population size is required to make the conclusion more convincing. When the data volume is large enough, we will conduct more detailed subgroup analysis and add other clinical variables to comprehensively analyze the diagnostic value of these markers in different stages, different locations and other clinical variables. In addition, right-sided colorectal cancer is said to have a poor prognosis. We can study how to improve the sensitivity of the detection of right-side colorectal cancer in the future. Third, this study focuses on the diagnostic value of mSEPT9 in CRC detection, yet the association between mSEPT9 and the prognosis of CRC patients is unknown. Fourth, we didn’t pay attention to the relationship in CRC patients between RAS, BRAF, MSI status, etc. and these markers. In the next step, we can collect the gene mutation status of CRC patients and do some relevant studies combined with these blood indicators to see if stronger identification of KRAS, BRAF, MSI status, etc. can be achieved. Therefore, large-scale and further prospective multicentre studies are needed to validate the clinical importance of mSEPT9 and its combined detection with other blood indicators in patients with CRC.

## Conclusion

In conclusion, we found in our study that plasma mSEPT9 represents a promising biomarker in CRC diagnosis. Notably, we discovered an important association between mSEPT9 status and the clinicopathological characteristics of patients with CRC. More importantly, we proved that the combination of mSEPT9, CEA, CA19-9 and PLR could significantly improve diagnostic efficacy in CRC.

## Data Availability

The authors declare that all data used and/or analysed during the current study are available from the corresponding author upon reasonable request.

## Abbreviations

mSEPT9: Methylated septin9 gene
CRC: Colorectal cancer
CEA: Carcinoembryonic antigen
CA19-9: Carbohydrate antigen 19 − 9
CA125: Carbohydrate antigen 125
PLT: Platelet
NLR: Neutrophil-lymphocyte ratio
PLR: Platelet-lymphocyte ratio
ROC: Receiver operating characteristic
AUC: Area under the ROC curve
